# Supplementary material for: Dexamethasone Suppression Testing in a Contemporary Cohort with Adrenal Incidentalomas in Two U.S. Integrated Healthcare Systems
Source: Biomedicines. 2023 Nov 28;11(12):3167. doi: 10.3390/biomedicines11123167 (PMC10740617; doi:10.3390/biomedicines11123167)
Supplement: Supplementary file 1 [file biomedicines-11-03167-s001.zip › biomedicines-2727857-supplementary.pdf]

Table S1. Autonomous diagnosis codes and electronic medical record problem list text used to identify adrenal incidentalomas.

Diagnostic Field	Description
**ICD-9 diagnostic code**	
227.0	Benign neoplasm of adrenal gland
237.2	Neoplasm of uncertain behavior of adrenal gland
255.8	Other specified disorders of adrenal glands
255.9	Unspecified disorder of adrenal glands
**ICD-10 diagnostic code**	
D35.0	Benign neoplasm of adrenal gland
D44.1	Neoplasm of uncertain behavior of adrenal gland
E27.8	Other specified disorders of adrenal gland
E27.9	Disorder of adrenal gland, unspecified
**Problem list from electronic medical record**	
Adenoma, bilat adrenal glands	
Adenoma, left adrenal gland	
Adenoma, right adrenal gland	
Adrenal incidentaloma	
Benign neoplasm, bilat adrenal glands	
Benign neoplasm, left adrenal gland	
Benign neoplasm, right adrenal gland	
Bilat adrenal mass	
Hx of adrenal adenoma	
Incidentaloma, adrenal gland.	
Left adrenal mass	
Right adrenal mass	
Neoplasm of uncertain behavior, bilat adrenal glands	
Neoplasm of uncertain behavior, left adrenal gland	
